# Identification and characterization of three *Vibrio alginolyticus* non-coding RNAs involved in adhesion, chemotaxis, and motility processes

**DOI:** 10.3389/fcimb.2015.00056

**Published:** 2015-07-10

**Authors:** Lixing Huang, Jiao Hu, Yongquan Su, Yingxue Qin, Wendi Kong, Ying Ma, Xiaojin Xu, Mao Lin, Qingpi Yan

**Affiliations:** ^1^Key Laboratory of Healthy Mariculture for the East China Sea, Ministry of Agriculture, Fisheries College, Jimei UniversityXiamen, China; ^2^College of Ocean and Earth Sciences, Xiamen UniversityXiamen, China

**Keywords:** ncRNA, *Vibrio alginolyticus*, adhesion, chemotaxis pathway, RNAi

## Abstract

The capability of *Vibrio alginolyticus* to adhere to fish mucus is a key virulence factor of the bacteria. Our previous research showed that stress conditions, such as Cu^2+^, Pb^2+^, Hg^2+^, and low pH, can reduce this adhesion ability. Non-coding (nc) RNAs play a crucial role in regulating bacterial gene expression, affecting the bacteria's pathogenicity. To investigate the mechanism(s) underlying the decline in adhesion ability caused by stressors, we combined high-throughput sequencing with computational techniques to detect stressed ncRNA dynamics. These approaches yielded three commonly altered ncRNAs that are predicted to regulate the bacterial chemotaxis pathway, which plays a key role in the adhesion process of bacteria. We hypothesized they play a key role in the adhesion process of *V. alginolyticus*. In this study, we validated the effects of these three ncRNAs on their predicted target genes and their role in the *V. alginolyticus* adhesion process with RNA interference (i), quantitative real-time polymerase chain reaction (qPCR), northern blot, capillary assay, and *in vitro* adhesion assays. The expression of these ncRNAs and their predicted target genes were confirmed by qPCR and northern blot, which reinforced the reliability of the sequencing data and the target prediction. Overexpression of these ncRNAs was capable of reducing the chemotactic and adhesion ability of *V. alginolyticus*, and the expression levels of their target genes were also significantly reduced. Our results indicated that these three ncRNAs: (1) are able to regulate the bacterial chemotaxis pathway, and (2) play a key role in the adhesion process of *V. alginolyticus*.

## Introduction

*Vibrio alginolyticus* is one of the most important opportunistic pathogens of marine fish, such as the cultured large yellow croaker, *Pseudosciaena crocea* (Kong et al., 2015). The ability of the bacterium to adhere to fish mucus is considered to be a key virulence factor of *V. alginolyticus* (Yan et al., 2007; Chen et al., 2008), and this ability could be affected by environmental conditions, although the molecular mechanism is unclear.

Bacterial non-coding (nc) RNAs are small regulatory RNAs (<500 nt). Although the conventional RNA interference (i) pathway has not yet been identified in the bacteria, bacterial non-coding (nc) RNAs have displayed several key roles in many biological processes by binding to the host's mRNA or protein targets (Livny et al., 2008; Cao et al., 2010). It has been proved that bacterial ncRNAs regulate responses to environmental stresses (Waters and Storz, 2009), including translation control, RNA degradation, and RNA processing. They have been shown to regulate a wide variety of biological processes, including secretion, quorum sensing, stress responses, biofilm formation, and virulence (Gottesman, 2005; Storz et al., 2005; Mann et al., 2012; Jorgensen et al., 2013). Some ncRNAs have also proved to play an important role in bacteria adhesion. For example, Mann et al. (2012) observed that DF20 and DF32/transfer messenger RNA could decrease adhesion to the nasopharyngeal or endothelial cells, respectively, of *Streptococcus pneumoniae*. OmpA protein is essential for adhesion of *Escherichia coli* to HeLa epithelial cells and Caco-2 colonic epithelial cells, while VrrA RNA has proved to affect *Vibrio cholerae* virulence by regulating the expression of OmpA and TcpA (Torres and Kaper, 2003; Song et al., 2008). In addition, VrrA is the first ncRNA proved to control the formation of outer membrane vesicles, which could promote adherence (Torres and Kaper, 2003; Song et al., 2008); however, the potential regulatory function of ncRNAs in *V. alginolyticus* adhesion remains unclear.

Our previous research showed that stress conditions such as Cu^2+^, Pb^2+^, Hg^2+^, and low pH are capable of reducing the adhesion ability of *V. alginolyticus* (Kong et al., 2015). We presented the first study of ncRNAs and genes (Kong et al., 2015) in *V. alginolyticus* cultured under normal and the above-mentioned stress conditions with RNA-seq. By doing this, we attempted to gain a broad spectrum of expression of potential ncRNAs and genes associated with bacterial adhesion. Target predictions and the Kyoto Encyclopedia of Genes and Genomes (KEGG) pathway analysis identified three significantly common altered ncRNAs predicted to regulate the bacterial chemotaxis pathway, which is closely allied to the movement and adherent capabilities of bacteria in response to a chemical stimulus (Bordas et al., 1998; Philippe et al., 2000; Victor and Tso, 2004; Mello and Tu, 2007; Takekawa et al., 2014).

Multiple transmembrane receptors, such as four methyl-accepting chemoreceptors (MCPs), are able to sense chemical gradients. These four receptors are methyl-esterified on conserved glutamate residues within their cytoplasmic domains by the methyl transferase CheR (Kleene et al., 1977; Springer and Koshland, 1977; Burgess-Cassler et al., 1982; Ahlgren and Ordal, 1983). Aer, a fifth receptor, is an aerotaxis, energy, and redox sensor (Bibikov et al., 1997, 2000; Rebbapragada et al., 1997; Repik et al., 2000). Binding a repellent at these receptors is believed to cause increased autophosphorylating activity of the CheA kinase (Bourret et al., 1991; Eisenbach, 1991). The phosphorylated form of CheA (CheA-P) phosphorylates CheY, which then interacts with the flagellar switch to cause clockwise (CW) rotation of the flagella. CheA-P could phosphorylate CheB, which in turn, demethylates the receptors. Demethylated receptors, with a repellent still bound to them, no longer augment CheA autophosphorylation, but rather maintain a level of CheA activity as is found in unstimulated cells. The importance of CheB in performing this function is underscored by the fact that mutants in cheB_E_ always tumble (Parkinson, 1976) because of a strong bias in CW flagellar rotation (Yonekawa et al., 1983). CheA-P also phosphorylates CheV, which could feed back to inhibit signal transduction from the receptors into the cytosol.

We hypothesized that these ncRNAs might play a key role in the adhesion process of *V. alginolyticus*. In the present study, we attempted to validate the effects of these three ncRNAs on their predicted target genes and the role of them in *V. alginolyticus* adhesion.

## Materials and methods

### Bacterial samples and culture conditions

Pathogenic *V. alginolyticus* (ND-01) was previously isolated from naturally infected *P. crocea* by our lab and confirmed as a pathogen by artificial infection (Kong et al., 2015). The sample was stored at −80°C in physiological saline with 10% glycerol. Bacteria were cultured on tryptic soy broth agar (TSA) supplemented with 2.0% NaCl at 28°C. Bacteria were challenged by the following chemical stresses: Cu^2+^ (50 mg/L CuSO_4_ ·5H_2_O), Pb^2+^ (100 mg/L (CH_3_COO)_2_Pb), Hg^2+^ (50 mg/L HgCl_2_), and low pH (HCl was used to lower pH to 5.0). It was presumed that these stresses could reduce the adhesion ability of *V. alginolyticus*. The control group was cultured in a normal TSA slant (pH = 7.0). Both of the stressed groups and the control group were analyzed with high-throughput sequencing, in order to detect stressed ncRNA dynamics. For validation of the results of high-throughput sequencing, the quantitative real-time (q) PCR was also performed on the stressed groups and the control group.

DH5α for GFP reporter gene analysis was bought from TransGen Biotech (China). Growth in Luria–Bertani (LB) broth (220 r.p.m., 37°C) or on LB plates at 37°C was used.

### Transient gene silencing

Transient gene silencing was used to investigate the function of the ncRNAs. 5'-Cy3-labeled short interfering (si) RNA comprising the predicted interaction sequence of ncRNA with a characteristic and highly specific 2–3-nucleotide 3' overhang that was synthesized by GenePharma Co. Ltd. (Shanghai, China). Negative control siRNA and treatment siRNA sequences are listed on Data Sheet 1.

*V. alginolyticus* strains were electrotransformed using a modified method established by Lancashire et al. (2005). To prepare competent cells, the culture of *V. alginolyticus* in the stationary phase was 1.0% (v/v) inoculated into 5.0 mL fresh lysogeny broth (LB) medium until an OD_600_ of 0.3–0.5 was reached. Bacteria were collected by centrifugation at 4000 g, for 10 min at 4.0°C and the pellet was washed twice in ice-cold sterile water and a third time in ice-cold 10% glycerol in water (v/v). Following the last centrifugation, the cells were resuspended in 1.0 mL 10% glycerol. Aliquots were frozen in dry ice and methanol and stored at −70°C.

Electroporation was performed using a Bio-Rad MicroPulser (Bio-Rad Laboratories, Inc., Hercules, CA, USA). Aliquots of 100 μL cell suspension were mixed with 2.0 μL siRNA (20 μM). Cells and siRNAs were transferred to a cuvette after being placed on ice for 30 min. Following electroporation (1.8 kV, 6.0 ms), 900 μL LB medium were immediately added and the cells were incubated at 28°C for 1.0, 6.0, 12, 24 h before RNA extraction and reverse-transcription polymerase chain reaction (RT-PCR). After incubating at 28°C for 6.0 h, cells were counterstained with 20 μg/mL DAPI (Roche Applied Science, USA). Images were taken using a fluorescence microscope (*n* = 6 per condition) to investigate the efficiency of uptake.

Because *V. alginolyticus* with RNA interference (i) treatments displayed significant silencing at 1.0–6.0 h (mentioned above), the *V. alginolyticus* cells were recovered at 28°C, 50 r/min for 2 h after electroporation to conduct the *in vitro* adhesion assay, as described below.

### Stable gene silencing

To further investigate the function of the ncRNAs, stable gene silencing was performed. Competent bacteria cells were collected using the methods mentioned above. The pACYC184 vector containing a short hairpin (sh) RNA (comprising the entire ncRNA sequence is listed in Table 1) and a non-target shRNA as a control (5'- TTC TCC GAA CGT GTC ACG TTT -3′) were used for stable gene silencing. The vector was transferred into SM10 by electrotransformation and then transferred by conjugation from SM10 to *V. alginolyticus*. Chloromycetin was used to screen the stable silenced clones, which were used for RNA extracts, capillary assay, and *in vitro* adhesion assay.

**Table 1:** **Non-coding (nc) RNAs used**.

**ncRNA: Candidate_907**
**Predicted target:** *cheB cheR mcp*
**ncRNA sequence:** UGGACUGGAAGGGCUGGAUCCAAAGCCUCAGUCUCUUCCACUUUCAUGAGUAAACCAUGUAAGGAAAGAUCUUGAAUCCUAGUUUCAAUUGCCAAGUCACGUUGUUCUACCUUCGCUGGCGCUUGAUGGAGAAUGCGUGAAAAGCGUCGUCUCUCUGCCAUGUGCAUUUUCUCGUCAUGUCUUCAAACAGAAUUGAAUAGUAUAUCAAUGCGAACAAAAAAUGUUCGAUUGCUGUUUGAAAAGUGUCGAGGUCGCUACAGAGUGACGUAAGUGCAAUGUCAUGUUCUUAACGAACGAGACGGGCUAAUGAG
**Interaction- ncRNA sequence (3'-5'):** *mcp*: CGUUGUUCUACCUUCGCUGGCGCUUGAUGGAGAAUGCGUGAAAAGCGUCG *cheB*: AGAUCUUGAAUCCUAGUUUCAAUUGCCAAGUCACGUUGUUCUACCUUC *cheR*: AGAUCUUGAAUCCUAGUUUCAAUUGCCAAGUCACGUUGUUCUACCUUC
**ncRNA: Candidate_431**
**Predicted target:** *mcp aer*
**ncRNA sequence:** CGGCUUUCUACUUCAGUGGUGAAUUCGUUGCUCUUAUCGGUGAGUAUAAACUGAAUUGUCGUAAUCGCAGAGUUUAGCUUGUCAGGAUCCGCACCAAGACUCAUUGGUAAGUUCAAUACCUCACCUGGCUGUACCUGAAUUGUCUGCUUACCAUACCAGCUUACAUCGGAUAGCCCUUCAACAUCCAACGAGUACUCUUGCACUUGCUGGGUUUUGUUGAUUACUUUGAGGGUGUAGGUGUUUUCUACUUCGCCCGAGCUAUUAACGCGGAACAGUUGGUUACGGUCGCGAAUAACACUCAUGCCGGCUGGGUCUACCGCUGCGAUUUGGGCAAAGAACAGACCAAUCAUGACCAGUAGCACAGCUCCAUAGCCCAGCAGCUUCGGACGCAUGACUUUGGUACUCUUACCCGAUAAGCGGUGCUCGGUGGUGUAGCUGAUUAGUCCUUUCUCGUAACCCAUGCGAUCCAUGGUGUUGUCACAUGCAUCAAUACAGGC
**Interaction- ncRNA sequence (3'-5'):** *mcp*: GGACGCAUGACUUUGGUACUCUUACCCGAUAAGCGGUGCUCGG *aer*: GGACGCAUGACUUUGGUACUCUUACCCGAUAAGCGGUGCUCGG
**ncRNA: Candidate_103**
**Predicted target:** *mcp cheV*
**ncRNA sequence:** AAGAAGAAUGAAAUGCUCAAAGGAUGAGUCCGUUUUAUCGCCUCCAAGGAACCGAGAAAUCAACGAAUGUUUAAUUGAUUUAAAAGGUGAUAGUUAUGAAAGGUUUACCAAGUACAAUGUUCUGGAAUAGCAAGUCUGUUUACACCGGCAACUUUGUAUACCCAACAAGCUUUGGCUACUAAGUGAAGUCAAAGCAUGUGACUCGAACAGUUUGUUCACCCCUAUAAAUUGGAAUUUUUUUUCAGUGAUAGUGCUGACAUGAUUCGCCGCCACCCAAAUUGGGUGGCGUUUUUUUAUCUCCACUUUCUCUUUCUGACACAUUUUCUGCCAUCAAGGCUGGAUUCUAUUCUCCGUUCUUCAGUUUUAUCGAC
**Interaction- ncRNA sequence (3'-5'):** *mcp*: CUCCACUUUCUCUUUCUGACACAUUUUCUGCCAUCAAGGCUGGAUUCUAUUCU *cheV*: UUUUUUUAUCUCCACUUUCUCUUUCUGACACAUUUUCUGCCAUCAAGGCUGGAUUCUAUUCUCCGUUC

### GFP reporter gene analysis

To validate the results of IntaRNA analysis, GFP reporter gene analysis was performed in *E. coli* using a method modified from other study (Corcoran et al., 2012). The pET28a-GFP vector containing the predicted interaction sequence of mRNA (Table 1) was transferred into DH5α by heat shock based transformation. Kanamycin was used to screen the positive clones. Then, the pACYC184 vector containing the entire ncRNA sequence we mentioned above was transferred into the positive clones by electrotransformation. Single bacterial colonies were screened with kanamycin and chloromycetin. The fluorescence was observed using a fluorescence microscope, and imaged with a digital video camera.

### Flow cytometric analysis

The flow cytometric analysis was performed using a method modified from other study (Corcoran et al., 2012). Single bacterial colonies were inoculated in 2 ml LB medium containing kanamycin and chloromycetin and grown at 37°C, 220 r.p.m. for 12 h. A culture volume corresponding to one OD600 per milliliter was mixed with 1 ml of phosphate-buffered saline (PBS) (pH 7.4) containing 4% paraformaldehyde and centrifuged for 2 min at 7500 g. Pellets were resuspended in 1 ml 1 × PBS. For flow cytometry analysis 1/1000 dilutions in 1 × PBS were prepared and measured on a CyFlow Space machine. Biological triplicates were prepared for every sample.

### RNA extraction and reverse transcription

Total RNA was extracted from the bacteria using TRIzol (Invitrogen, Carlsbad, CA, USA) according to the manufacturer's protocol. First-strand complementary (c) DNA was synthesized from 2.0 mg total RNA using a Revert Aid Mu-MLV cDNA synthesis kit (TransGen Biotech, China) according to the manufacturer's protocol.

### Northern blot analysis

For northern blot analysis of ncRNAs and the predicted target genes, typically 20 mg total RNA were loaded into each lane, and ribosomal RNA bands were visualized using ethidium bromide staining. The 5'-biotin-labeled oligo nucleotides used as probes are listed on Data Sheet 2. The probes were detected on the membranes by streptavidin-Alexa Fluor 680 conjugate (Invitrogen, Carlsbad, CA, USA) according to the manufacturer's instructions.

### Quantitative real-time polymerase chain reaction

Expression levels of ncRNAs and the predicted target genes were verified by qPCR using the Power SYBR Green PCR Master Mix (AppliedBiosystems, USA) according to the manufacturer's instructions, and normalized with *16srna* (which showed an invariant expression under the experimental conditions) calculated by means of the 2^−ΔΔCt^ method (*n* = 6). Primers are listed in Data Sheet 3.

### Mucus preparation

Healthy *P. crocea* were obtained from marine cage cultures, Ningde, Fujian Province, China. Skin mucus was prepared using a method modified from a previous study (Kong et al., 2015). The fish were washed with sterile phosphate buffered saline (PBS) (0.01 mol/L, pH 7.2). The skin mucus was harvested by scrapping the surface of the skin with a plastic spatula to remove the mucus gel layer covering the skin; the mucus gel was then homogenized in PBS. The mucus preparations were centrifuged twice at 20,000 g and 4.0°C for 30 min to remove particulate materials. The final supernatant was filtered through 0.45- and 0.22-μm pore filters. The mucus samples were adjusted to 1.0 mg protein/mL PBS. The protein concentration was determined using the method of Bradford (1976).

### Capillary assay

The capillary assay was used to study bacterial chemotaxis according to Zaval' skii et al. (2003). A capillary tube with an inner diameter of 0.1 mm was sealed at one end and filled with the mucus. The tube was dipped into a bacterial suspension containing approximately 10^8^ CFU/mL. After incubating for 1.0 h, the chemotaxis of bacterial cells was evaluated from a comparison of the numbers of cells penetrating into this tube and into the negative control tube, which was filled with buffer containing no mucus. The number of bacterial cells in the capillary tubes was accurately determined by plating the contents of the tubes onto agar media. Three trials of each group were conducted.

### *In vitro* adhesion assay

The bacterial adhesion assay was conducted following the method described by Yan et al. (2006). Briefly, 50 μL mucus suspension were spread evenly onto 22 mm^2^ glass slides and fixed with methanol for 20 min after the mucus was dry. Then, 1.0 mL aliquots of bacterial suspensions (10^8^ CFU/mL) was placed on the mucus-coated glass slides, incubated moistly at 25°C for 2.0 h, and washed thoroughly five times with PBS. Finally, the slides were fixed with 4.0% methanol for 30 min, dyed with crystal violet for 3.0 min, and bacteria were counted under a microscope (×1000). Three trials were conducted for each group and 20 microscope visions were selected.

### Data analyses

Results were reported as means ± S.D. The data were statistically analyzed with One-Way ANOVA followed by Dunnett's multiple comparison tests using SPSS 13.0 (SPSS Inc. Chicago, IL, USA). A value of *P* < 0.05 was used to indicate a significant difference.

## Results

### Validation of the results of high-throughput sequencing

**Figure 1:**
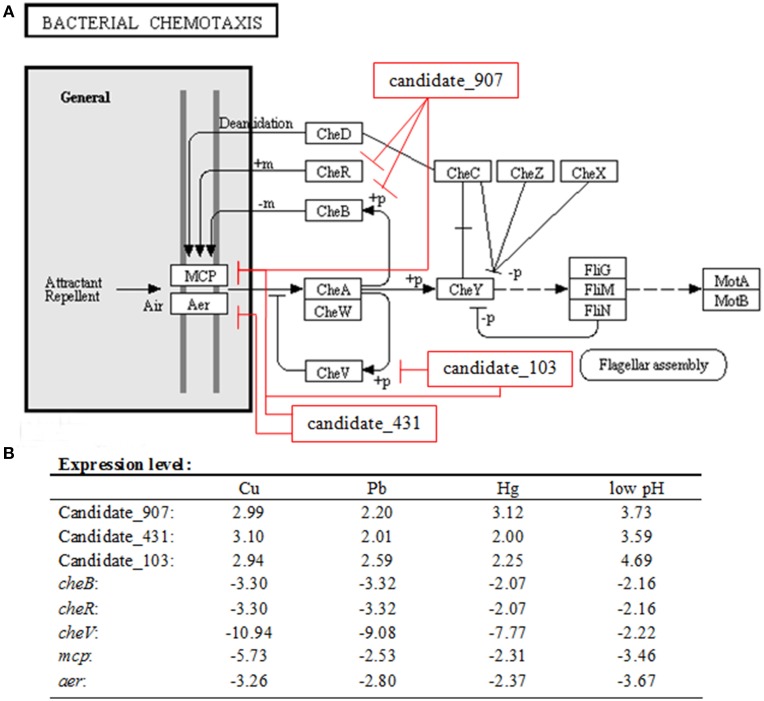
**Overall scheme for predicated non-coding RNAs involved in the bacterial chemotaxis pathway. (A)** *cheB*, *cheR*, *cheV*, *mcp*, and *aer* are the key regulators of the bacterial chemotaxis pathway. MCP is able to sense chemical gradients and methyl-esterified on conserved glutamate residues within their cytoplasmic domains by the methyl transferase CheR. Aer is an aerotaxis, energy, and redox sensor. Binding a repellent at these receptors is believed to cause increased autophosphorylating activity of the CheA kinase. The phosphorylated form of CheA (CheA-P) phosphorylates CheY, which then interacts with the flagellar switch to cause clockwise (CW) rotation of the flagella. CheA-P could phosphorylate CheB, which in turn, demethylates the receptors. Demethylated receptors, with a repellent still bound to them, no longer augment CheA autophosphorylation, but rather maintain a level of CheA activity as is found in unstimulated cells. CheA-P also phosphorylates CheV, which could feed back to inhibit signal transduction from the receptors into the cytosol. We found three ncRNAs significantly changed by Cu^2+^, Pb^2+^, Hg^2+^, and low pH simultaneously, which was predicted to regulate the bacterial chemotaxis pathway: Candidate_907 was predicted to inhibit the expression of *mcp*, *cheR*, and *cheB*; Candidate_103 was predicted to inhibit the expression of *mcp* and *cheV*; Candidate_431 was predicted to inhibit the expression of *mcp* and *aer*. **(B)** Expression level of ncRNAs and predicted target genes detected by high-throughput sequencing.

The effects of various environmental stresses (Cu^2+^, Pb^2+^, Hg^2+^, low pH, high pH, low salinity, high salinity, low temperature, and high temperature) on *V. alginolyticus* adhesion were detected in our previous research. Our results indicated that Cu^2+^, Pb^2+^, Hg^2+^, and low pH could reduce the adhesion ability of *V. alginolyticus*; therefore, the expressions of ncRNAs and genes in *V. alginolyticus* cultured under normal and stress conditions (Cu^2+^, Pb^2+^, Hg^2+^, and low pH with RNA-seq) were compared. The data were deposited in the National Center for Biotechnology Information Sequence Read Archive, which can be accessed with accession number SRP049226. IntaRNA was used to predict interactions between significantly changed ncRNA and mRNA, after which the target genes were used for gene ontology and KEGG pathway analysis. These analyses yielded three ncRNAs significantly changed by Cu^2+^, Pb^2+^, Hg^2+^, and low pH simultaneously, which was predicted to regulate the bacterial chemotaxis pathway (Figure 1A). Because the bacterial chemotaxis pathway is closely related to bacterial adhesion, this indicated that the ncRNAs might play an important role in *V. alginolyticus* adhesion to host species. The predicted target genes, ncRNA sequence, and interaction sequence of ncRNA detected by high-throughput sequencing are listed in Table 1. According to the results of high-throughput sequencing, these three ncRNAs were significantly up-regulated by Cu^2+^, Pb^2+^, Hg^2+^, and low pH simultaneously, while their target genes were all significantly down-regulated. Cu^2+^, Pb^2+^, Hg^2+^, and low pH treatment significantly up regulated the expression of Candidate_103 (by 2.94-, 2.59-, 2.25-, and 4.69-fold), Candidate_431 (by 3.10-, 2.01-, 2.00-, and 3.59-fold), and Candidate_907 (by 2.99-, 2.20-, 3.12-, and 3.73-fold) (Figure 1B). Cu^2+^, Pb^2+^, Hg^2+^, and low pH treatment significantly reduced the expression of *cheB* (by 3.30-, 3.32-, 2.07-, and 2.16-fold), *cheR* (by 3.30-, 3.32-, 2.07-, and 2.16-fold), *mcp* (by 5.73-, 2.53-, 2.31-, and 3.46-fold), *aer* (by 3.26-, 2.80-, 2.37-, and 3.67-fold), and *cheV* (by 10.94-, 9.08-, 7.77-, and 2.22-fold) (Figure 1B). This indicated that these ncRNAs can negatively control the expression of *cheB*, *cheR*, *cheV*, *mcp*, and *aer*, which are the key regulators of the bacterial chemotaxis pathway.

**Figure 2:**
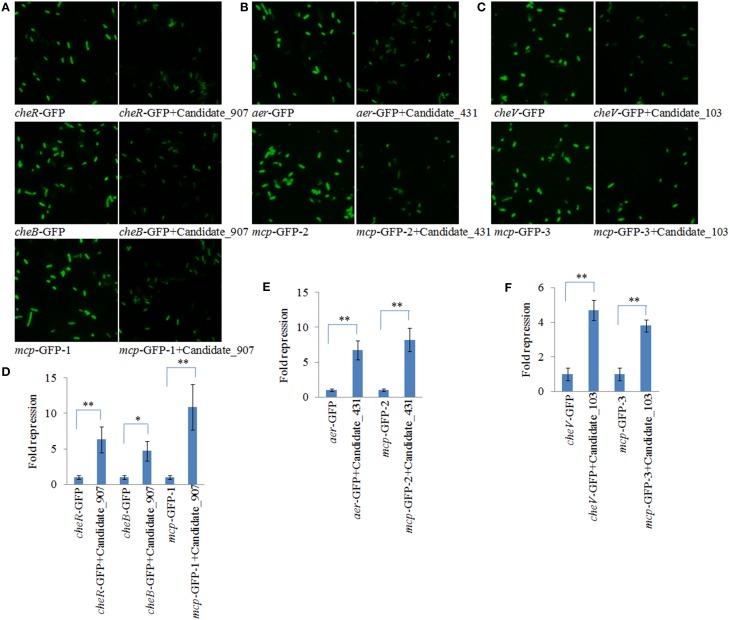
**GFP reporter gene analysis revealed interactions between the ncRNAs and the mRNA of target genes. (A–C)** Fluorescence intensities of *E. coli* containing target gene-GFP plasmid and vectors with a shRNA comprising the entire ncRNA sequences under fluorescence microscope. GFP fluorescence was excited at 460 nm, and light emission was recorded using a 510 nm filter. **(D–F)** Flow cytometric analysis of target regulation. Fold repression is depicted on the *y*-axis. Data are presented as the mean ± *SD* (*n* = 3, ^**^P < 0.01, ^*^P < 0.05).

To validate the results of IntaRNA analysis, GFP reporter gene analysis was performed in *E. coli*. Under the fluorescence microscope, *E. coli* containing target gene-GFP plasmids displayed green fluorescence, while the fluorescence intensities were significantly repressed by vectors containing a shRNA comprising the entire ncRNA sequences (Figures 2A–C). The results of flow cytometric analysis also showed that the ncRNA sequences were sufficient for repression of the target genes by 4.71- to 10.93-fold (Figures 2D–F). These results reinforced the prediction of IntaRNA, and indicated the interactions between the ncRNAs and the mRNA of target genes. Anyway, further research is still necessary.

**Figure 3:**
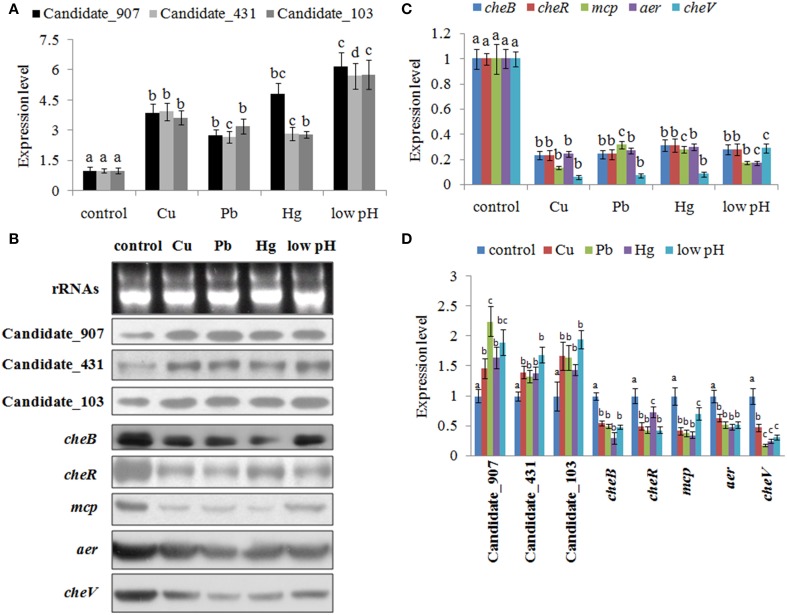
**Environmental stresses induce the expression of non-coding (nc) RNAs and inhibit the expression of their target genes. (A)** Quantitative real-time polymerase chain reaction (qPCR) analysis of the expression of ncRNAs (including Candidate_907, Candidate_431, and Candidate_103) after stress treatments. Data are presented as mean ± S.D. (*n* = 6). Means of treatments not sharing a common letter are significantly different at *P* < 0.05. **(B)** The ncRNAs and their target genes were detected by northern blot (lower panel). The ribosomal (r) RNA bands stained with ethidium bromide (upper panel) were used as loading controls. **(C)** qPCR analysis of the target genes (*cheB*, *cheR*, *cheV*, *mcp*, and *aer*) after stress treatments. Data are presented as the mean ± S.D. (*n* = 6). Means of treatments not sharing a common letter are significantly different at *P* < 0.05. **(D)** Intensities of northern blot bands were quantified using densitometry. Results are expressed as multiples (× fold) of optical density of target bands and the rRNA determined in the control. The mean expression from the control was designated as 1 in the graph. Values (mean ± S.D.) are representative of data obtained in three independent experiments (*n* = 3). Treatments not sharing a common letter are significantly different at *P* < 0.05 as assessed by One-Way ANOVA followed by the Dunnett's test.

To validate the results of RNA-seq, qPCR was performed on these three ncRNAs and their predicted target genes. The results of qPCR matched those of the sequencing—Cu^2+^, Pb^2+^, Hg^2+^, and low pH treatment significantly up regulated the expression of Candidate_103 (by 3.65-, 3.21-, 2.79-, and 5.77-fold), Candidate_431 (by 3.94-, 2.67-, 2.84-, and 5.71-fold), and Candidate_907 (by 3.89-, 2.75-, 4.84-, and 6.19-fold) (Figure 3A), while the expression of their predicted target genes was significantly down regulated by all treatments (Figure 3C). Cu^2+^, Pb^2+^, Hg^2+^, and low pH treatment significantly reduced the expression of *cheB* (by 4.29-, 4.15-, 3.21-, and 3.59-fold), *cheR* (by 4.28-, 4.15-, 3.21-, and 3.60-fold), *mcp* (by 7.45-, 3.16-, 3.58-, and 5.74-fold), *aer* (by 4.14-, 3.72-, 3.37-, and 5.84-fold), and *cheV* (by 16.96-, 14.07-, 12.04-, and 3.44-fold).

To further validate these results, northern blot was performed and intensities of northern blot bands were quantified using densitometry. The results of northern blot showed that stresses could significantly induce the expression of these ncRNAs and reduce the expression of their predicted target genes (Figures 3B,D). Cu^2+^, Pb^2+^, Hg^2+^, and low pH treatment significantly up regulated the expression of Candidate_103 (by 1.67-, 1.64-, 1.43-, and 1.94-fold), Candidate_431 (by 1.40-, 1.33-, 1.39-, and 1.69-fold), and Candidate_907 (by 1.46-, 2.24-, 1.64-, and 1.90-fold). Cu^2+^, Pb^2+^, Hg^2+^, and low pH treatment significantly reduced the expression of *cheB* (by 1.82-, 2.01-, 3.36-, and 2.04-fold), *cheR* (by 2.00-, 2.30-, 1.36-, and 2.31-fold), *mcp* (by 2.37-, 2.58-, 2.84-, and 1.42-fold), *aer* (by 1.56-, 1.92-, 2.08-, and 1.92-fold), and *cheV* (by 2.10-, 5.50-, 4.00-, and 3.14-fold).

These results reinforced the reliability of our sequencing data, indicating that these ncRNAs and their predicted target genes were sensitive to environmental stresses. Also, these results indicated that these ncRNAs can negatively control the expression of *cheB*, *cheR*, *cheV*, *mcp*, and *aer*, which are the key regulators of the bacterial chemotaxis pathway.

### Effects of transient gene silencing

**Figure 4:**
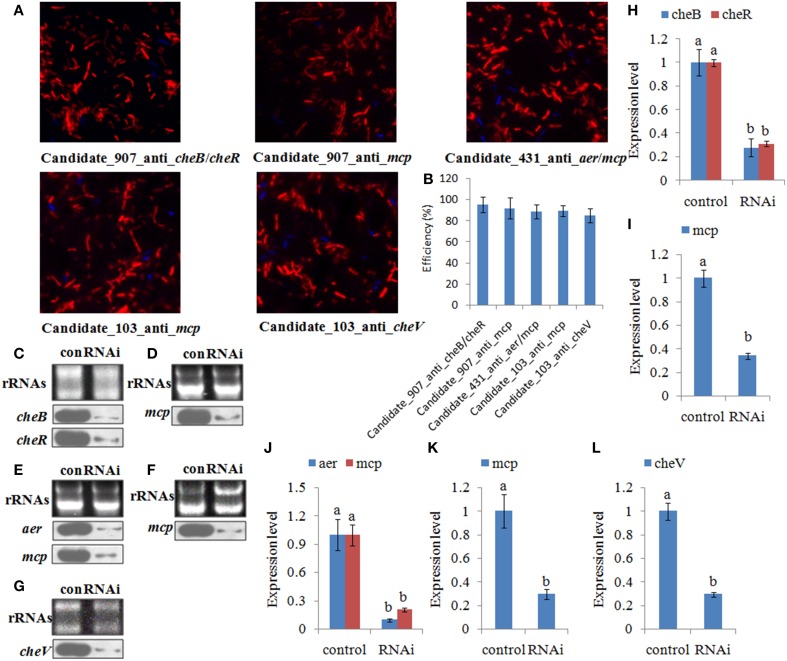
**The efficiency of short interfering (si) RNA uptake. (A,B)** Representative overlay images of Cy3 (red) and DAPI (blue) under fluorescence microscopy displayed the uptake of siRNA. Quantification of uptake efficiency was presented as the mean ± S.D. (*n* = 6). **(C–G)** The expression of the target genes were detected by northern blot (lower panel) at 1.0 h after treating with siRNAs. The rRNA bands stained with ethidium bromide (upper panel) were used as loading controls. **(H–L)** Intensities of northern blot bands were quantified using densitometry. Results are expressed as multiples (× fold) of optical density of target bands and the rRNA determined in the control. The mean expression from the control was designated as 1 in the graph. Values (mean ± S.D.) are representative of data obtained in three independent experiments (*n* = 3). Treatments not sharing a common letter are significantly different at *P* < 0.05 as assessed by One-Way ANOVA followed by the Dunnett's test.

The efficiency of siRNA uptake was detected under fluorescence microscopy 6.0 h after electrotransformation. Red fluorescence indicated that the siRNA could be delivered into the *V. alginolyticus* by electrotransformation (Figure 4A). Our results showed a high uptake efficiency of 85–95% (Figure 4B). Northern blot showed that siRNA treatment could significantly reduce the expression of their predicted target genes at 1.0 h after treating with siRNAs (Figures 4C–G). Intensities of northern blot bands were quantified using densitometry, which showed that the target genes were significantly reduced by 1.36- to 10.26- fold at 1.0 h (Figures 4H–L).

**Figure 5:**
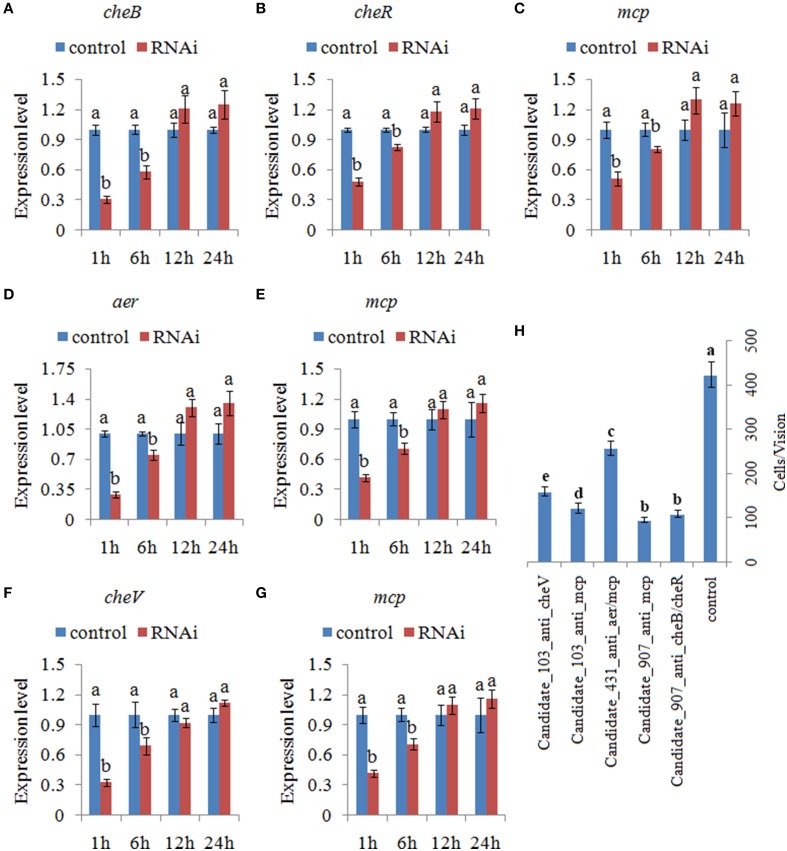
**Transient gene silencing reduced the expression of the target genes and the adhesion ability of *V. alginolyticus***. Quantitative real-time polymerase chain reaction (qPCR) analysis of the expression of **(A)** *cheB*, **(B)** *cheR*, and **(C)** *mcp* after transient gene silencing with Candidate_907_anti_*cheB*/*cheR* and Candidate_907_anti_*mcp* at 1.0, 6.0, 12, and 24 h. qPCR analysis of the expression of **(D)** *aer* and **(E)** *mcp* after transient gene silencing with Candidate_431_anti_*aer*/*mcp* at 1.0, 6.0, 12, and 24 h. qPCR analysis of the expression of **(F)** *cheV* and **(G)** *mcp* after transient gene silencing with Candidate_103_anti_*cheV* and Candidate_103_anti_*mcp* at 1.0, 6.0, 12, and 24 h. Data are presented as the mean ± S.D. (*n* = 6). Means of treatments not sharing a common letter are significantly different at *P* < 0.05. **(H)** The capacity of transient silenced *V. alginolyticus* to adhere to mucus. Data are presented as the mean ± S.D. (*n* = 3). Means of treatments not sharing a common letter are significantly different at *P* < 0.05, as assessed using One-Way ANOVA followed by the Dunnett's test.

After treating with siRNAs, the expression of the target genes was also detected at 1.0, 6.0, 12, and 24 h. The expression levels of these target genes with siRNA treatments were normalized against the corresponding control (scrambled) siRNA treatments. The target genes were significantly reduced by 1.21- to 3.45-fold at 1.0–6.0 h. After 6.0 h, the target genes were not significantly changed compared to those of the control. Based on the percent decrease in expression (Figures 5A–G), *V. alginolyticus* with siRNA treatments displayed significant (*p* < 0.05) gene silencing at 1.0–6.0 h, while siRNAs were not efficient in *V. alginolyticus* at any of the time points after 6.0 h. The reduction in target genes means that the predicted interaction sequences of ncRNAs were true, which indicates that a system similar to the conventional RNAi pathway might exist in *V. alginolyticus*, although the mechanism remains unclear. Also, these results further proved that these ncRNAs can negatively control the expression of *cheB*, *cheR*, *cheV*, *mcp*, and *aer*.

The ability of *V. alginolyticus* to adhere to the host under normal and RNAi conditions was then compared at 2.0 h. Our results showed that approximately 423 cells/vision control *V. alginolyticus* adhered to the slides, while the numbers of adherent bacterial cells of Candidate_907_anti_cheB/cheR-, Candidate_907_anti_mcp-, Candidate_431_anti_aer/mcp-, Candidate_103_anti_cheV-, and Candidate_103_anti_mcp-RNAi *V. alginolyticus* were 108, 95, 256, 121, and 158 cells/vision, respectively (Figure 5H). This means the adhesion ability of *V. alginolyticus* was reduced by 3.92-, 4.45-, 1.65-, 3.50-, and 2.68-fold after RNAi, which demonstrates that the adhesion ability of *V. alginolyticus* under RNAi conditions is significantly impaired. Thus, these ncRNAs together with their target genes might play an important role in *V. alginolyticus* adhesion to host species.

### Effects of stable gene silencing

**Figure 6:**
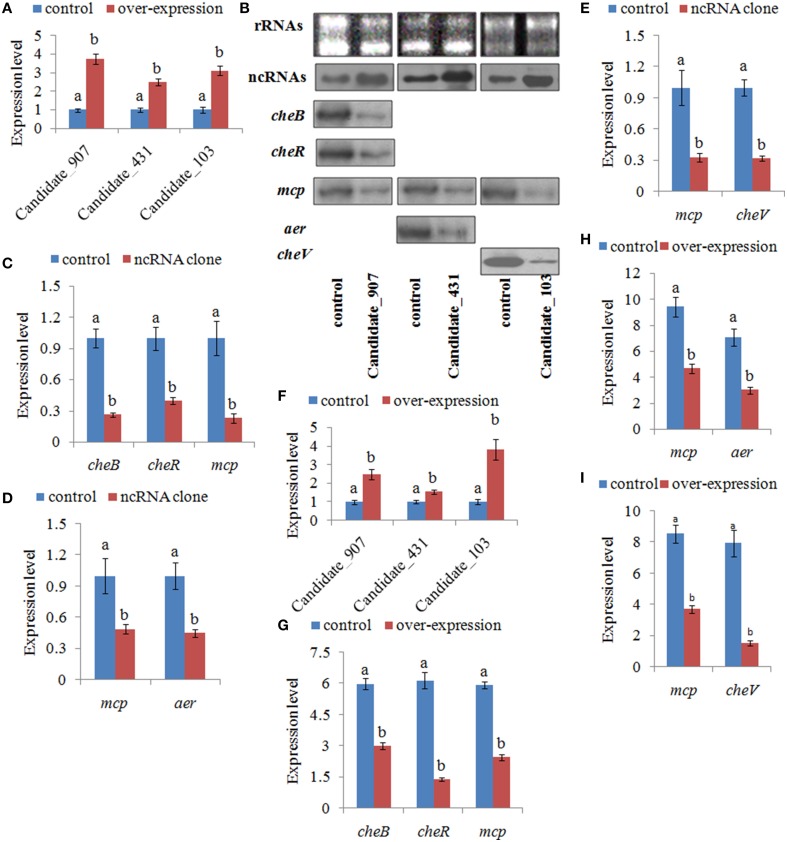
**Stable gene silencing reduced the expression of the target genes. (A)** Quantitative real-time polymerase chain reaction (qPCR) analysis of the expression of non-coding (nc) RNAs (Candidate_907, Candidate_431, and Candidate_103) in stable silenced clones. Data are presented as the mean ± S.D. (*n* = 6). Means of treatments not sharing a common letter are significantly different at *P* < 0.05. **(B)** The ncRNAs and their target genes were detected by northern blot (lower panel). The rRNA bands stained with ethidium bromide (upper panel) were used as loading controls. **(C)** qPCR analysis of the expression of *cheB*, *cheR*, and *mcp* after stable gene silencing with Candidate_907. **(D)** qPCR analysis of the expression of *mcp* and *aer* after stable gene silencing with Candidate_431. **(E)** qPCR analysis of the expression of *mcp* and *cheV* after stable gene silencing with Candidate_103. Data are presented as the mean ± S.D. (*n* = 6). Means of treatments not sharing a common letter are significantly different at *P* < 0.05. **(F–I)** Intensities of northern blot bands were quantified using densitometry. Results are expressed as multiples (× fold) of optical density of target bands and the rRNA determined in the control. The mean expression from the control was designated as 1 in the graph. Values (mean ± S.D.) are representative of data obtained in three independent experiments (*n* = 3). Treatments not sharing a common letter are significantly different at *P* < 0.05 as assessed by One-Way ANOVA followed by the Dunnett's test.

To further validate the function of these ncRNAs and the role they play in regulating adhesion, stable gene silencing was performed with a vector containing a shRNA comprising the entire ncRNA sequence. Our results of qPCR showed that the expression levels of ncRNAs in stable silenced clones are significantly increased by 3.76-, 2.51-, and 3.13-fold, respectively (Figure 6A). The results of northern blot also showed that ncRNAs are significantly increased in stable silenced clones (Figure 6B). Intensities of northern blot bands were quantified using densitometry, which showed that the ncRNAs were significantly increased by 2.47-, 1.54-, and 3.82-fold, respectively (Figure 6F). These results reinforce the reliability of stable gene silencing. The expression level of the target genes was also detected. The results of qPCR showed that the target genes are significantly reduced in stable silenced clones by 2.04- to 4.35-fold (Figures 6C–E). The results of northern blot also showed that the target genes are significantly reduced in stable silenced clones by 2.00- to 5.12-fold (Figures 6B,G–I). The reduction of target genes indicates that the entire ncRNA sequence could decrease the expression of their target genes.

**Figure 7:**
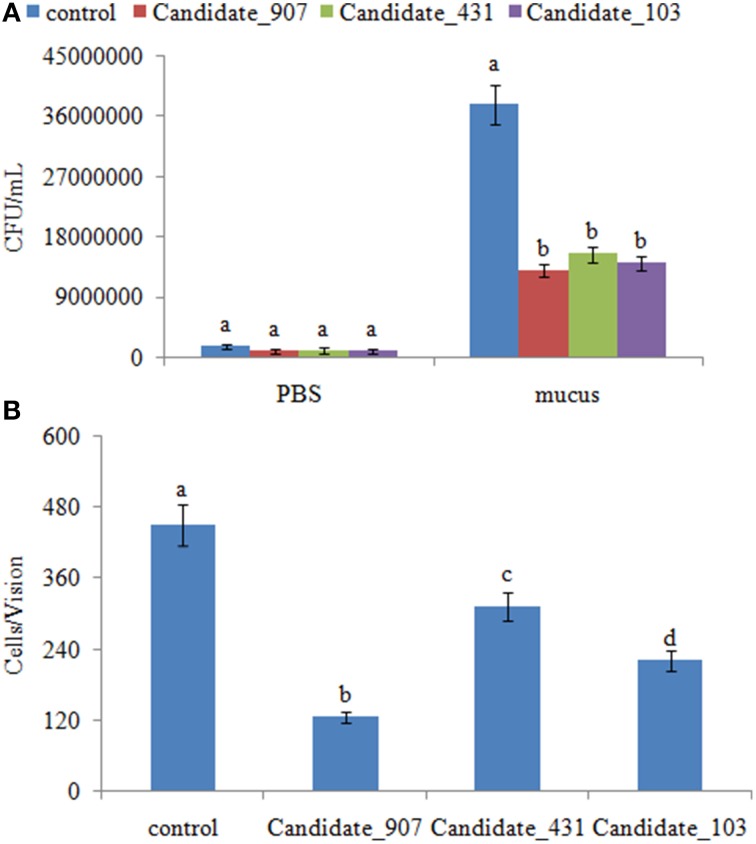
**Stable gene silencing reduced the chemotaxis capacity and adhesion capacity of *V. alginolyticus* to mucus. (A)** The chemotaxis capacity of stable silenced *V. alginolyticus*. Data are presented as the mean ± S.D. (*n* = 3). Means of treatments not sharing a common letter are significantly different at *P* < 0.05, as assessed using One-Way ANOVA followed by the Dunnett's test. **(B)** The capacity of stable silenced *V. alginolyticus* to adhere to mucus. Data are presented as the mean ± S.D. (*n* = 3). Means of treatments not sharing a common letter are significantly different at *P* < 0.05, as assessed using One-Way ANOVA followed by the Dunnett's test.

Because the target genes of these ncRNAs belong to the bacterial chemotaxis pathway, it was hypothesized that these ncRNAs might affect adhesion by perturbing the chemotaxis of *V. alginolyticus*. To validate this hypothesis, the chemotaxis of stable silenced clones was detected. Our results showed that the chemotactic ability of stable silenced clones is significantly impaired. The chemotactic ability of 907-, 431-, and 103-RNAi *V. alginolyticus* was reduced by 2.91-, 2.45-, and 2.70-fold, respectively (Figure 7). This indicated that these ncRNAs can negatively control the chemotaxis of *V. alginolyticus* by negatively controlling the expression of *cheB*, *cheR*, *cheV*, *mcp*, and *aer*, which are the key regulators of the bacterial chemotaxis pathway.

The adhesion ability of stable silenced clones was also detected. Our results showed that approximately 450 cells/vision control *V. alginolyticus* adhered to the slides, while the numbers of adherent bacteria of 907-, 431-, and 103-RNAi *V. alginolyticus* were 125, 313, and 220 cells/vision, respectively (Figure 7). This means the adhesion ability of *V. alginolyticus* was reduced by 3.60-, 1.44-, and 2.05-fold in stable silenced clones, which also demonstrates that the adhesion ability of stable silenced clones is significantly impaired. This further proves that these ncRNAs can negatively control the adhesion process of *V. alginolyticus* by negatively controlling the transcription of *cheB*, *cheR*, *cheV*, *mcp*, and *aer*.

## Discussion

New developments in sequencing technologies have resulted in exponential increases in our understanding of bacterial ncRNA (Mann et al., 2012). Although several kinds of ncRNAs have been identified in bacteria, none were specifically implicated in *V. alginolyticus* adhesion. In addition, no attempts have been made to apply RNAi to investigate the role of ncRNAs in bacterial adhesion. Our research represents the first use of RNAi to investigate the role of ncRNAs in the process of adhesion based on RNA-seq.

The bacterial chemotaxis pathway is essential for bacterial colonization, which means that decreased chemotaxis ability leads to decreased attachment onto the host (Milner and Sellwood, 1994; Yao and Allen, 2006). *cheB*, *cheR*, *cheV*, *mcp*, and *aer* are key regulators of the bacterial chemotaxis pathway (Yao and Allen, 2006), and mutation of these genes leads to a reduction in bacterial chemotaxis (Watts et al., 2004; Garvis et al., 2009; Lowenthal et al., 2009; Zhang et al., 2013; Lambert et al., 2015). In this study, three new *V. alginolyticus* ncRNAs were found that regulate the expression of *cheB*, *cheR*, *cheV*, *mcp*, and *aer*. According to our results, these ncRNAs are direct negative regulators of *cheB*, *cheR*, *cheV*, *mcp*, and *aer* mRNA. Our results also indicated that these ncRNAs might regulate the *V. alginolyticus* chemotaxis and adhesion processes; therefore, an increase of these ncRNAs leads to a reduction in *V. alginolyticus* chemotactic and adhesion ability by negatively controlling the transcription of *cheB*, *cheR*, *cheV*, *mcp*, and *aer*. Nevertheless, although IntaRNA predicted that there are interactions between the ncRNAs and the mRNA of target genes, which was supported by GFP reporter gene analysis, further research is necessary to elucidate how the ncRNAs inhibit these target genes.

RNA-mediated regulation is quite different from protein-mediated regulation, which might have a number of potential advantages for bacteria (Beisel and Storz, 2010). First, ncRNAs do not require translation and occupy much less of the genome. Second, ncRNAs can have multiple targets, while multiple ncRNAs can regulate a single target under different conditions (Repoila et al., 2003; Waters and Storz, 2009). Finally, ncRNAs have dramatically different half-lives, ranging from 2.0 to >30 min (Vogel et al., 2003). These differences could potentially affect the duration of regulation mediated by ncRNAs. The challenge that remains after identification of ncRNAs in bacteria is to assign them discrete functional roles (Mann et al., 2012). Our results displayed the feasibility of addressing the role of ncRNAs in the process of adhesion with RNAi based on RNA-seq, which suggests that the combination of RNA-seq and RNAi is a powerful tool for further research on the roles of these ncRNAs in *V. alginolyticus*.

In summary, this study demonstrated that these ncRNAs negatively control the adhesion process of *V. alginolyticus* by negatively controlling the expression of *cheB*, *cheR*, *cheV*, *mcp*, and *aer*, which are the key regulators of the bacterial chemotaxis pathway. To our knowledge, this is the first such study about how *V. alginolyticus* ncRNAs can affect the bacteria's virulence. Because these ncRNAs repress rather than promote virulence in *V. alginolyticus*, adhesion repression by some methods affecting the expression of ncRNAs could be considered the basis of a strategy for therapeutic intervention in the pathogenicity of *V. alginolyticus*.

### Conflict of interest statement

The authors declare that the research was conducted in the absence of any commercial or financial relationships that could be construed as a potential conflict of interest.
