# Supplementary material for: Identification and characterization of three Vibrio alginolyticus non-coding RNAs involved in adhesion, chemotaxis, and motility processes
Source: Front Cell Infect Microbiol. 2015 Jul 10;5:56. doi: 10.3389/fcimb.2015.00056 (PMC4498440; doi:10.3389/fcimb.2015.00056)
Supplement: Supplementary file 1 [file Table1.DOCX]

**Table S1. siRNA Sequence**

| **Name** | **siRNA Sequence (5’-3’)** |
| --- | --- |
| Candidate_907_anti_*cheB*/*cheR* | F: AGAUCUUGAAUCCUAGUUUCAAUUGCCAAGUCACGUUGUUCUACCUUCTT  R: GAAGGUAGAACAACGUGACUUGGCAAUUGAAACUAGGAUUCAAGAUCUTT |
| Candidate_907_anti_*mcp* | F: CGUUGUUCUACCUUCGCUGGCGCUUGAUGGAGAAUGCGUGAAAAGCGUCGTT  R: CGACGCUUUUCACGCAUUCUCCAUCAAGCGCCAGCGAAGGUAGAACAACGTT |
| Candidate_431_anti_*aer*/*mcp* | F: GGACGCAUGACUUUGGUACUCUUACCCGAUAAGCGGUGCUCGGTT  R: CCGAGCACCGCUUAUCGGGUAAGAGUACCAAAGUCAUGCGUCCTT |
| Candidate_103_anti_*mcp* | F: CUCCACUUUCUCUUUCUGACACAUUUUCUGCCAUCAAGGCUGGAUUCUAUUCUTT  R: AGAAUAGAAUCCAGCCUUGAUGGCAGAAAAUGUGUCAGAAAGAGAAAGUGGAGTT |
| Candidate_103_anti_*cheV* | F: UUUUUUUAUCUCCACUUUCUCUUUCUGACACAUUUUCUGCCAUCAAGGCUGGAUUCUAUUCUCCGUUCTT  R: GAACGGAGAAUAGAAUCCAGCCUUGAUGGCAGAAAAUGUGUCAGAAAGAGAAAGUGGAGAUAAAAAAATT |
| Negative control | F: UUCUCCGAACGUGUCACGUTT  R: ACGUGACACGUUCGGAGAATT |
