# Supplementary material for: Identification and characterization of three Vibrio alginolyticus non-coding RNAs involved in adhesion, chemotaxis, and motility processes
Source: Front Cell Infect Microbiol. 2015 Jul 10;5:56. doi: 10.3389/fcimb.2015.00056 (PMC4498440; doi:10.3389/fcimb.2015.00056)
Supplement: Supplementary file 2 [file Table2.DOCX]

**Table S2. Probes for northern blot analysis**

| **ncRNA** | **Primers for reverse transcription** |
| --- | --- |
| Candidate_103 | 5'-CTTGGAGGCGATAAAACGGACTCATCCTTTGAGCATTTCATTCTTCTT-3' |
| Candidate_431 | 5'-TATACTCACCGATAAGAGCAACGAATTCACCACTGAAGTAGAAAGCCG-3' |
| Candidate_907 | 5'-CATGAAAGTGGAAGAGACTGAGGCTTTGGATCCAGCCCTTCCAGTCCA-3' |
| *cheB* | 5'-GTAAACCTGGTCCTGATAGAAAATATGTTCGCCAATGTCTTTTGCCAC-3' |
| *cheR* | 5'-TCTTTAATGGCAGACCAATGTAAGCTTGAATACCATCCTCTTGAAACA-3' |
| *cheV* | 5'-AAAGCCAATTATCCAACCAAAATACGCATCAGAAAAAGTTTGATGCAA-3' |
| *mcp* | 5'-TAGCAGCAAAAATGACGAGGCTGTGACAACCTTATGTCTAAATTTCAT-3' |
| *aer* | 5'-CTCCCTCACGAGAGTTTCTTGAGCTTTATGAGAAATATTGACTGACAT-3' |
