# Supplementary material for: Identification and characterization of three Vibrio alginolyticus non-coding RNAs involved in adhesion, chemotaxis, and motility processes
Source: Front Cell Infect Microbiol. 2015 Jul 10;5:56. doi: 10.3389/fcimb.2015.00056 (PMC4498440; doi:10.3389/fcimb.2015.00056)
Supplement: Supplementary file 3 [file Table3.DOCX]

**Table S3. Primers for QPCR**

| **ncRNA** | **Primers for reverse transcription** | |
| --- | --- | --- |
| Candidate_103 | 5'-GTCGTATCCAGTGCAGGGTCCGAGGTATTCGCACTGGATACGACATAGAGG-3' | |
| Candidate_431 | 5'-GTCGTATCCAGTGCAGGGTCCGAGGTATTCGCACTGGATACGACTGCACGA-3' | |
| Candidate_907 | 5'-GTCGTATCCAGTGCAGGGTCCGAGGTATTCGCACTGGATACGACCAGTGCA-3' | |
| **ncRNA/gene** | **Primers for QPCR** | |
| Candidate_103 | 5'-TACCGTCTTTTACACAGTCTTT-3' (F) | 5'-GCAGGGTCCGAGGTATTC-3' (R) |
| Candidate_431 | 5'-GGCCTATATCGATGCCGGTAA -3' (F) | 5'-GCAGGGTCCGAGGTATTC-3' (R) |
| Candidate_907 | 5'-GTACCGTCTCTCTGCTGCGA -3' (F) | 5'-GCAGGGTCCGAGGTATTC-3' (R) |
| *mcp* | 5'-GGTTGAGGGCTCTTCTGTGGTC-3' (F) | 5'-TGTACGAACTTCATCAGCAACGAC-3' (R) |
| *aer* | 5'- TGAACAAATCCAAGCGTCTATCTCT-3' (F) | 5'- GCTCTACGGAGTGCGTTGCT-3' (R) |
| *cheB* | 5'- GGCAGGCTGGACGAACTATC-3' (F) | 5'- GAACTTGGCAATGCTTGTGGA-3' (R) |
| *cheR* | 5'- GCAGGCTGGACGAACTATCG-3' (F) | 5'- AACTTGGCAATGCTTGTGGA-3' (R) |
| *cheV* | 5'- TCATCGCGGATATTAACCCA-3' (F) | 5'- GATCACATTGTAACCTGAAGAAGCT-3' (R) |
| *16s rna* | 5'- GGGGAGTACGGTCGCAAGAT-3' (F) | 5'- CGCTGGCAAACAAGGATAAGG-3' (R) |
